# Psychiatric Hospitalizations of People Found Not Criminally Responsible on Account of Mental Disorder in France: A Ten-Year Retrospective Study (2011–2020)

**DOI:** 10.3389/fpsyt.2022.812790

**Published:** 2022-04-05

**Authors:** Thomas Fovet, Maëlle Baillet, Mathilde Horn, Christine Chan-Chee, Olivier Cottencin, Pierre Thomas, Guillaume Vaiva, Fabien D'Hondt, Ali Amad, Antoine Lamer

**Affiliations:** ^1^Univ. Lille, Inserm, CHU Lille, U1172 - Lille Neuroscience & Cognition, Lille, France; ^2^Psychiatry and Addiction Medicine Department, CHU Lille, Lille, France; ^3^Centre national de ressources et de résilience Lille-Paris (CN2R), Lille, France; ^4^Univ. Lille, Faculté Ingénierie et Management de la Santé, Lille, France; ^5^Univ. Lille, CHU Lille, ULR 2694 - METRICS: Évaluation des Technologies de santé et des Pratiques médicales, Lille, France; ^6^Santé publique France, Saint Maurice cedex, France

**Keywords:** forensic—psychiatric practice, prison, France, not criminally responsible on account of mental disorder, responsibility, insanity defense, not guilty by reason of insanity

## Abstract

**Background:**

Criminal responsibility is a key concept in the criminal sanctioning of people diagnosed with mental health disorders who have committed crimes. In France, based on the recommendations of one or more expert psychiatrists, a judge can declare a person not criminally responsible on account of mental disorder (NCRMD) if, at the time of the offense, the person was presenting a psychiatric disorder that abolished or altered his/her capacity for discernment and/or ability to control his/her actions. In such a case, the judge also generally orders an involuntary psychiatric hospitalization. The objectives of this study were to (1) describe longitudinal retrospective administrative data of psychiatric hospitalizations for people found NCRMD, (2) identify the age, sex, and principal diagnoses of these individuals, and (3) characterize the trajectories of their psychiatric care before and after NCRMD psychiatric hospitalization.

**Methods:**

We used discharge reports from the French national hospital database called *Programme de médicalisation des systèmes d'information* (PMSI) to gather longitudinal data that describe psychiatric hospitalizations for people found NCRMD between 2011 and 2020, the age, sex, and principal diagnoses of these patients, the length of their hospitalization, and the trajectories of their psychiatric care before and after their NCRMD psychiatric hospitalization.

**Results:**

We identified 3,020 patients who were hospitalized for psychiatric care after having been found NCRMD between 2011 and 2020. The number of admissions on these grounds has remained stable over this period, ranging from 263 in 2011 to 227 in 2021. They were mostly young men diagnosed with a psychotic disorder (62%). The majority (87%) were hospitalized in general psychiatric hospitals, and only 13% were admitted to maximum-security units (*Unités pour malades difficiles, UMD*). The median duration of hospitalization for these patients was 13 months. Our results show that 73% of the patients had already been hospitalized prior to their NRCMD hospitalization. The rehospitalization rate within 5 years of discharge from NCRMD psychiatric hospitalization was 62%.

**Conclusion:**

We conducted the first study investigating the psychiatric hospital treatment of people declared NCRMD in France. There is an urgent need for further studies to investigate the clinical characteristics of these patients.

## Introduction

The treatment of people diagnosed with mental disorders who commit crimes differs greatly in countries around the world (1). This is mainly due to the specific histories of criminal justice and psychiatry in each country (2, 3). Despite the well-documented epidemiological and service-level differences regarding the management of these people in Europe (4), it remains that in many countries, criminal responsibility, whose origin dates back to the Bablylonian Talmud (written around 500 AD), Roman law, and Greek philosophy (5), is a key concept in the criminal sanctioning of people diagnosed with mental health disorders (6, 7).

In France, criminal responsibility has been a core principle of criminal law since the early nineteenth century (8). Three categories of criminal responsibility are currently considered: (i) lack of criminal responsibility (“*A person who was suffering, at the time of the offense, from a psychic or neuropsychic disorder that abolished his/her ability to control his/her actions”*), (ii) diminished criminal responsibility (“*A person who was suffering, at the time of the offense, from a psychic or neuropsychic disorder that impaired his/her ability to control his/her actions*”) and (iii) full criminal responsibility. To decide on the level of responsibility a person who has committed a crime bears, the judge or the court may defer to the recommendations of one or more expert psychiatrist.

The expert's assessment must include whether the committed offense was a direct result of a mental health disorder that abolished or altered the offender's capacity for discernment and/or ability to control his/her actions according to article 122–1 of French criminal law. If an expert concludes that there was a lack of criminal responsibility, the judge can declare the person not criminally responsible on account of mental disorder (NCRMD) and thus not subject to a prison sentence (*Law of February 25th 2008*). The lack of criminal responsibility is then registered in the criminal record. Importantly, the judge can impose security measures, such as prohibiting access to victims or family members or restrictions regarding the perpetrator's place of residence and/or freedom to travel. In almost all cases, the judge also orders an involuntary hospitalization in a general psychiatric hospital or in a maximum-security psychiatric unit (called *unité pour malades difficiles*, UMD) if the person “*endangers the safety of others and [needs] care, supervision and safety measures [that] can only be carried out in a specific unit*”, according to a specific law (article L3213–7 of *Public Health Code*). In France, there are a total of 10 UMDs (620 beds for men, 36 beds for women) fully managed by the public health system and therefore totally independent from the justice system.

Although in the last quarter of the twentieth century there has been a decrease reported in the number of people declared NCRMD in France (9), no recent data are available on the number of people found to have been hospitalized on the grounds of NCRMD. Of note, deinstitutionalization has been associated with an increase in the number of people found NCRMD as well as a rise in forensic beds in several countries (10–13). Despite the significant implications from a public health perspective (14), the sociodemographic and clinical characteristics of these persons remain completely unknown in France, as does the average duration of the hospitalizations. Similarly, the psychiatric care trajectories before and after NCRMD psychiatric hospitalization have never been investigated, nor has the impact of a stay in a maximum-security psychiatric unit (UMD), which is a major issue for this population (15).

The present study used discharge reports from the French national hospital database called *Programme de médicalisation des systèmes d'information* (PMSI) for the period from 2011 to 2020 to describe longitudinal retrospective administrative data of psychiatric hospitalizations for people found NCRMD. We also aimed to describe the age, sex, and principal diagnoses of these individuals, the length of hospitalization, and the trajectories of their psychiatric care before and after NCRMD psychiatric hospitalization.

## Methods

### Data Source

The PMSI database, which contains standardized discharge reports for all inpatient stays in psychiatric hospitals in France, was used in this study. A unique national identification number for each patient allows the data from all hospital stays for the same patient to be linked. The database includes individual-level data on the dates of admission and discharge, hospital code number, sector code, and outcome (i.e., discharge, hospital transfer, death). Sociodemographic data from these records include sex, age, and place of residence. The principal diagnosis, defined as the main reason for admission, and the associated diagnoses related to comorbidities, are also collected and coded according to the French version of the International Statistical Classification of Diseases and Related Health Problems, 10th Revision (ICD10). The following criterion allowed us to identify relevant cases in the PMSI: “specific legal mode used for the hospitalization of people found NCRMD (*Mode Légal 4*)”.

### Study Population

We identified all the psychiatric hospitalizations for people found NCRMD in France between 2011 and 2020. All patients concerned were included in the current study.

### Variables

For all the psychiatric hospitalizations for people found NCRMD, we collected the age and sex of the patient, hospital stay/duration, discharge status (death or not), principal diagnosis, and type of facility (general psychiatric hospital or maximum-security psychiatric unit). We identified stays in maximum-security psychiatric units (UMDs) with the letter “D” in the hospitalization category. We also collected information on the year of admission when patients were admitted before 2011.

The following categories of principal diagnosis were used: (F1) mental and behavioral disorders due to psychoactive substance use, (F2) schizophrenia, schizotypal, delusional, and other non-mood psychotic disorders, (F3) mood (affective) disorders, (F4) anxiety, dissociative, stress-related, somatoform and other nonpsychotic mental disorders, (F6) disorders of adult personality and behavior, (F7) intellectual disabilities, and other disorders.

### Statistical Analysis

For each year, we present the number of ongoing psychiatric hospitalizations of people found NCRMD, as well as the number of admissions to and discharges from NCRMD psychiatric hospitalization. Age on admission, sex, principal diagnosis on admission, duration of hospitalization, and death before discharge are described for each patient.

As no data were available before 2011, we divided the study population into two groups to study the trajectories before NCRMD admission and after NCRMD hospitalization discharge:

*Group 1* consisted of patients found NCRMD who were admitted in or after 2016, allowing us to study their psychiatric care trajectory over the 5 years before their NCRMD admission. For *Group 1*, we examined whether patients with prior hospitalizations had the same distribution of principal diagnoses as those without prior hospitalizations. When patients had already been hospitalized before their NCRMD admission, we investigated whether the principal diagnosis of the NCRMD hospitalization was already documented in at least one of the hospitalizations during the 5 previous years.

*Group 2* consisted of patients discharged from an NCRMD psychiatric hospitalization before 2016. For *Group 2*, we computed the delay between discharge and the subsequent hospitalization, allowing us to study the psychiatric care trajectory over the 5 years after the NCRMD discharge. We examined the rate of rehospitalization according to whether the patient had been hospitalized in a UMD.

Quantitative variables are described as the median (1st quartile−3rd quartile). The normality of the distributions was checked graphically and by applying the Shapiro–Wilk test. Qualitative variables are described as the frequency (percentage). For inter-group comparisons, we used Student's *t*-test or the Mann-Whitney-Wilcoxon test for quantitative variables or the chi-squared test or Fisher's exact test for qualitative variables, depending on the data distribution and the sample size.

### Ethics

French legislation does not require the approval of an independent ethics committee for retrospective registry-based studies. The data extraction and analysis of the study was approved by the *Commission Nationale de l'Informatique et des Libertés* (CNIL; authorization number: 1754053).

## Results

### Number of Patients NCRMD Hospitalized

**Figure 1:**
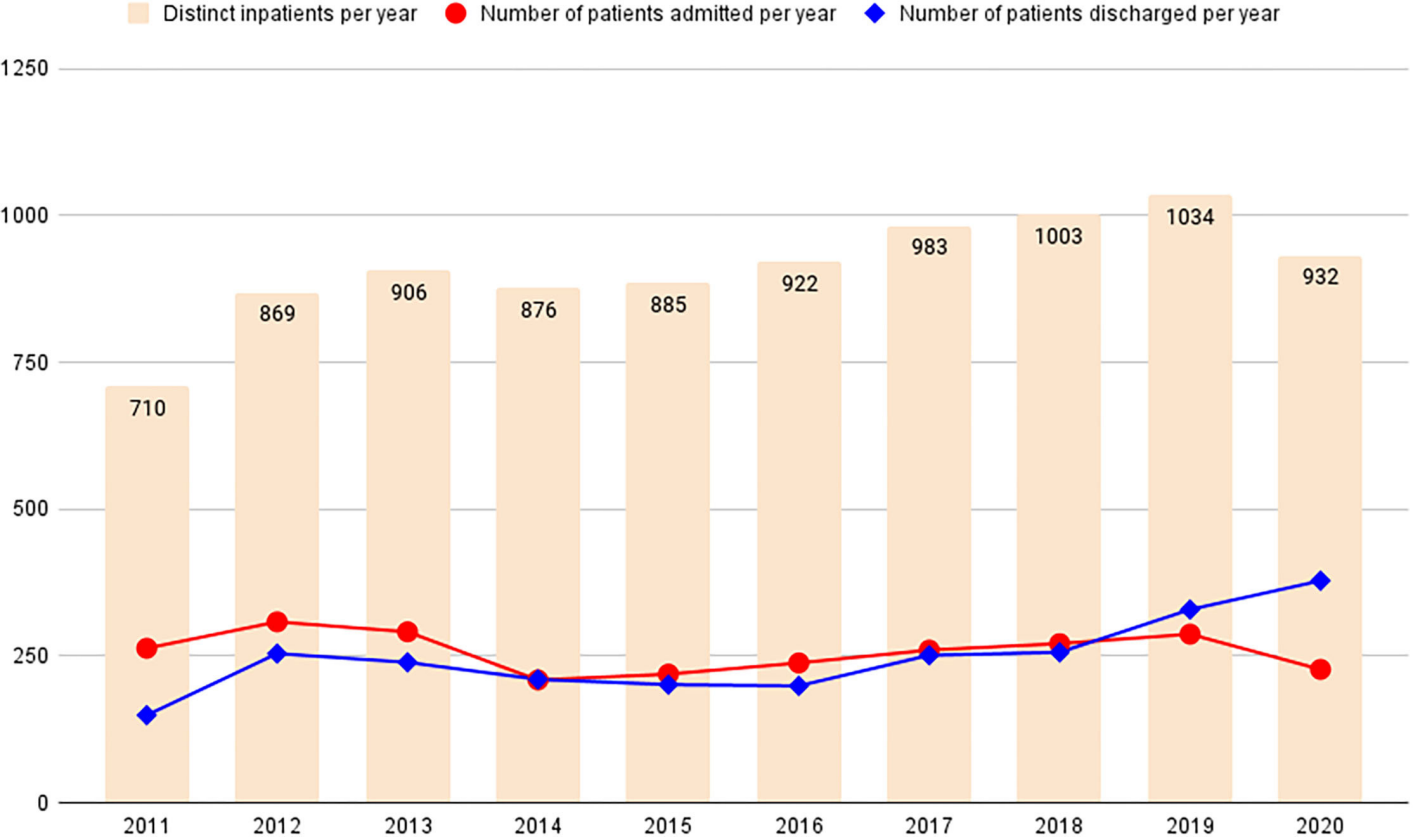
Number of individuals found not criminally responsible on account of mental disorder (NCRMD) and referred to psychiatric hospitalization in France (2011–2020).

Between 2011 and 2020, 3020 patients were hospitalized for psychiatric care after having been found NCRMD. The total number of hospitalized patients in any single year ranged from a minimum of 710 in 2011 to a maximum of 1,034 in 2019. The number of admissions and discharges per year ranged from a minimum of 209 to a maximum of 308 and from a minimum of 149 to a maximum of 378, respectively. Four hundred and forty-seven patients were admitted before 2011, with the earliest admission in 1975. Overall, 2,573 patients were admitted and 2,466 patients were discharged during the study period (between 2011 and 2020). Figure 1 represents the total number of patients found NCRMD who were hospitalized, admitted, and discharged each year (see also Supplementary Material).

### Age, Sex and Diagnoses

The demographic characteristics and main diagnoses of the 3,020 distinct patients found NCRMD who were referred to psychiatric hospitalization are presented in Table 1. Of these, 393 (13%) had a stay in a maximum-security psychiatric unit (UMD). These patients were younger, more often male, and had a slightly different diagnostic profile than those who were not referred to a stay in a UMD (see Table 1).

**Table 1:** Demographic characteristics and diagnoses of individuals found not criminally responsible on account of mental disorder and referred to psychiatric hospitalization in France (2011–2020).

	**Total (*N* = 3020)**	**Without UMD stay (*N* = 2627)**	**With UMD stay (N = 393)**
Age, years (on admission or in 2011 for patients already hospitalized)	36 (28;45)	36 (28;46)	33 (27;41)
Sex—Male	2680 (88.8%)	2297 (87.5%)	383 (97.5%)
Diagnosis			
*F2—Schizophrenia, schizotypal, delusional, and other non-mood psychotic disorders*	1867 (61.8%)	1556 (59.2%)	311 (79.1%)
*F6 - Disorders of adult personality and behavior*	286 (9.5%)	250 (9.6%)	36 (9.2%)
*F3—Mood (affective) disorders*	183 (6.1%)	179 (6.8%)	4 (1.0%)
*F1—Mental and behavioral disorders due to psychoactive substance use*	146 (4.8%)	139 (5.3%)	7 (1.8%)
*F4–Anxiety, dissociative, stress-related, somatoform and other nonpsychotic mental disorders*	80 (2.6%)	80 (3.0%)	0 (0.0%)
*F7—Intellectual disabilities*	56 (1.9%)	49 (1.9%)	7 (1.8%)
*Other disorders*	254 (8.4%)	234 (8,9%)	20 (5.1%)
*Missing diagnosis*	148 (4.9%)	140 (5.3%)	8 (2.0%)

*p <0.001 for age (Student's t-test), sex (chi-squared test), and diagnosis (chi-squared test)*.

### Duration of Hospitalization

The median duration of hospitalization for the overall sample was 13 [2;48] months. For the 2466 patients discharged between 2011 and 2020, the median duration of hospitalization was 9 (1;35) months. For the 554 patients still hospitalized in 2020, the median duration of hospitalization was 50 (19;101) months. The duration of hospitalization was longer for patients hospitalized in UMDs (see Table 2).

**Table 2:** Duration of hospitalization (in months) and death during hospitalization for patients found not criminally responsible on account of mental disorder and referred to psychiatric hospitalization in France (2011–2020).

	**Total (*N* = 3020)**	**No UMD (*N* = 2627)**	**UMD (*N* = 393)**	***p* value**
Duration of stay—median (Q1–Q3)	13 (2;48)	10 (2;38)	49 (21;87)	<0.001
Duration of completed stay—median (Q1–Q3)	9 (1;35)	7 (1;30)	36 (14;74)	<0.001
Duration of current stay—median (Q1–Q3)	50 (19;101)	42 (14;96)	66 (36;107)	0.006
Death—n, %	41 (1.4%)	33 (1.3%)	8 (2.0%)	0.213

Overall, 41 patients (40 men; 1 woman) died during the NCRMD hospitalization. The median frequency of death was 3.5 (min: 1, max: 9) per year, and the median age at death was 45 (33;59) years. Death occurred after 5 (1;8) years of hospitalization.

### Psychiatric Care Pathway Before Admission and After Discharge

**Figure 2:**
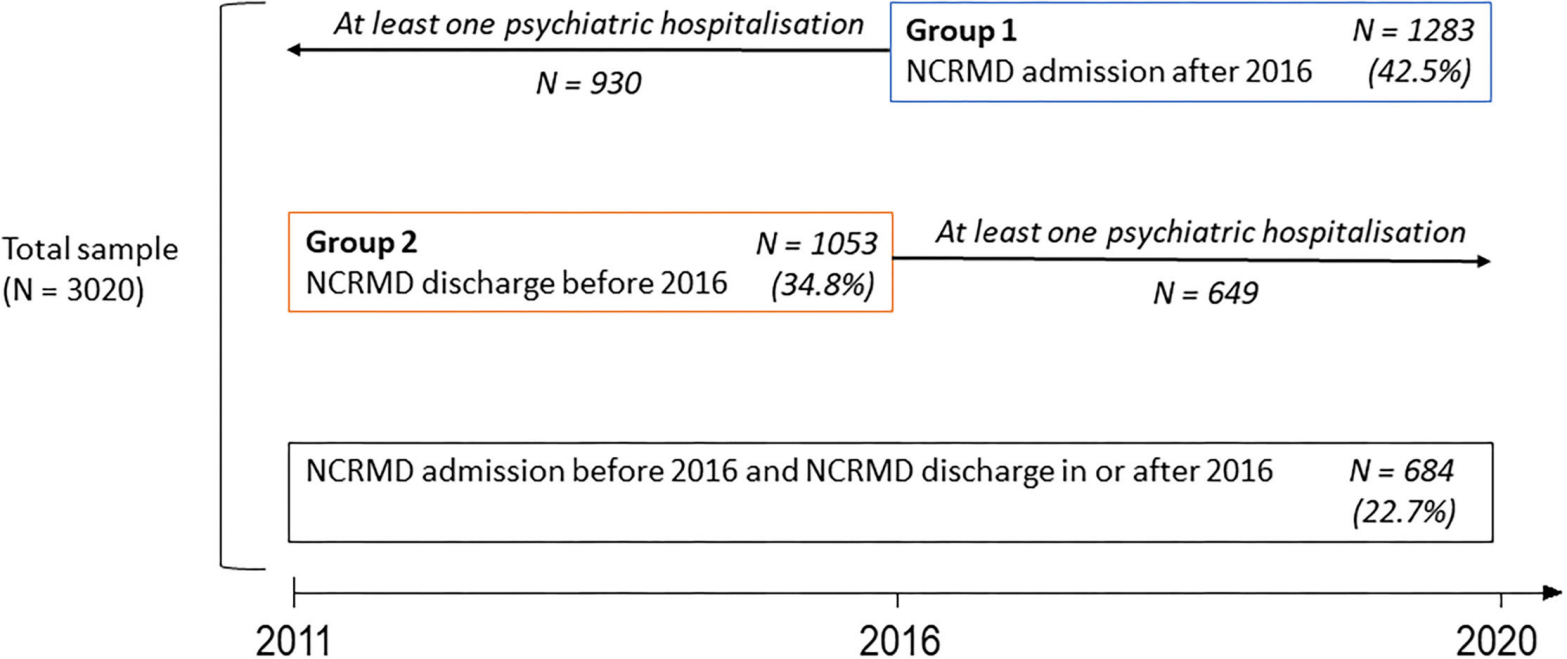
Characterization of the sample of people found not criminally responsible on account of mental disorder in France (2011–2020).

The sample sizes for Groups 1 and 2 are shown in Figure 2.

#### Psychiatric Care Pathway Before Admission

We focused on patients who were admitted in or after 2016 (Group 1) to investigate the psychiatric care pathways during the 5 years prior to their admission on the grounds of NCRMD. Among these 1,283 patients, 930 (72.5%) had already been hospitalized in the five previous years, including 879 patients for whom a principal diagnosis was documented. For 75 patients (8.3%), at least one of these prior hospitalizations had taken place in a UMD. Importantly, for 730 patients (84% of the total number of patients with a documented diagnosis in both hospitalizations), the diagnosis that motivated hospitalization for NCRMD had already been identified during a previous hospitalization. The concordance between the diagnoses of the two hospitalizations is presented in Table 3.

**Table 3:** Principal diagnoses that motivated the hospitalization for lack of criminal responsibility and psychiatric hospitalizations during the previous 5 years for patients found not criminally responsible on account of mental disorder admitted after 2016 in France (*n* = 879).

	**Principal diagnosis that motivated the hospitalization for lack of criminal responsibility**	**Principal diagnosis that motivated at least one previous hospitalization**	**Concordance**
*F2—Schizophrenia, schizotypal, delusional, and other non-mood psychotic disorders*	565	513	90.8%
*F6—Disorders of adult personality and behavior*	80	55	68.7%
*F3—Mood (affective) disorders*	67	54	80.6%
*F1—Mental and behavioral disorders due to psychoactive substance use*	52	36	69.2%
*F4—Anxiety, dissociative, stress-related, somatoform and other non-psychotic mental disorders*	29	23	79.3%
*Other disorders*	88	49	55.7%
Total	879	730	83.0%

The principal diagnoses identified for NCRMD hospitalization among the 353 patients without any prior psychiatric hospitalization differed significantly from those with at least one prior hospitalization (see Supplementary Material). Notably, patients with prior hospitalization more often had a psychotic disorder diagnosis than those without prior hospitalization (64.6% vs. 54.1%, respectively; *p* = 0.004).

#### Psychiatric Care Pathway After Discharge

We focused on patients found NCRMD who were discharged before 2016 (Group 2) to study the psychiatric care pathway during the 5 years after discharge. Among the 1,053 patients discharged before 2016, 649 (61.6%) were readmitted to a general psychiatric hospital or a UMD (for 28 patients) in the 5 years following discharge. The mean delay between the end of their hospitalization due to a lack of criminal responsibility and the subsequent hospitalization was 141 (5–507) days. Of the 649 patients who were rehospitalized, 52 (8.0%) had been hospitalized in a UMD for their initial NCRMD stay, whereas only 15 (3.7%) of the 404 patients who were not rehospitalized after discharge (p = 0.008) had been hospitalized in a UMD for their initial NCRMD stay.

## Discussion

To the best of our knowledge, the present study is the first to investigate the psychiatric hospital treatment of people found NCRMD in France. We identified 3,020 patients who were referred to psychiatric hospital care after having been found NCRMD between 2011 and 2020 in France. They were mostly young men with a diagnosis of psychotic disorder, which is in line with previous research conducted in other countries (16, 17). Most of these patients (87%) were admitted to a general psychiatric hospital, and 13% were admitted to a maximum-security unit (UMD). The median duration of hospitalization for these patients was 13 months.

A decrease in the number of people found NCRMD in the last quarter of the twentieth century in France has been reported (9). This phenomenon results in people with severe psychiatric disorders who have committed offenses being referred to prison rather than to psychiatric facilities. This is often considered one of the major causes of the high prevalence of psychiatric disorders observed in French correctional facilities (18–20). Our results indicate that this trend did not continue between 2011 and 2020, as the number of people found NCRMD has remained stable over this period, ranging from 263 in 2011 to 227 in 2021, with a minimum of 209 in 2014 and a maximum of 308 in 2012.

Between 2011 and 2020, the number of patients found NCRMD who were treated in psychiatric services increased from 710 in 2011 to 932 in 2020, with a maximum of 1,034 in 2019. This increase can be explained by the lower number of patients discharged (*n* = 2466) compared to the number of patients admitted (*n* = 2573) during this period and the relatively long length of stay for this type of hospitalization. This proportional increase in forensic patients present in the general psychiatric system has been observed in many European countries and is referred to by some authors as “*forensification*" (21). This situation raises several questions regarding the management of individuals found NCRMD. First, these patients pose specific clinical challenges, such as the management of risk for violence in the hospital setting or complex psychiatric presentations (22). General psychiatric hospitals are therefore not always adequate for the optimal care of this population. Second, as shown in our study, the length of hospitalization of these patients is long, which appears to be poorly compatible with current health policies that tend to reduce the number of general psychiatric beds available in most countries (23). The deinstitutionalisation of general psychiatric hospitals has been accompanied by the development of forensic psychiatric services in several countries across Europe (24), a phenomenon called re-institutionalization by some authors (25), but no facilities are specifically designed to accommodate people found NCRMD in France (26).

In France, maximum-security units, called UMDs, can admit patients who “endanger the safety of others and for whom the necessary care, supervision and safety measures can only be carried out in a specific unit” (27). We found that only a small proportion of the individuals found NCRMD (13%) were admitted to UMDs. These patients present certain sociodemographic and clinical characteristics that differ from those of individuals found NCRMD who are admitted to general psychiatric hospitals: they are more often men, they are on average younger, and they more often have a diagnosis of psychotic disorder (79 vs. 59%). They also have a longer median length of hospitalization (49 months vs. 10 months for patients without a UMD stay), and they are more likely to be re-hospitalized within 5 years after NCRMD discharge, which suggests that these patients present the most complex and severe clinical presentations (28). Importantly, many studies have shown that diagnostic comorbidity of a psychotic disorder with a personality disorder, substance use disorders and neurodevelopmental disorders are highly prevalent in people admitted to forensic psychiatric settings (29, 30).

For the entire study population, we measured a rehospitalization rate of 62% within 5 years of discharge from their NCRMD psychiatric hospitalization, which is in line with previous studies conducted in other countries (31, 32). It should be noted that, unlike in other countries, individuals found NRCMD cannot be subject to long-term judicial oversight in France. Only involuntary outpatient treatment can be established at the end of a full-time involuntary psychiatric hospitalization (33). This kind of program is not court-ordered and can only be decided by the treating psychiatrist. If the person does not comply with the outpatient care, he/she may be readmitted to the hospital.

Regarding pre-admission management, our results show that 73% of the patients had already been hospitalized before their NRCMD stay, which is consistent with previous research. A Canadian longitudinal study involving 1,800 men and women found NCRMD reported that 72.4% of the study sample had at least one prior psychiatric hospitalization (17). Importantly, we found that in 83% of the cases, the diagnosis related to the NRCMD stay had been correctly identified during the previous hospitalization. The correspondence was particularly high for psychotic disorders (91%). This result provides a particularly interesting perspective in terms of prevention and raises the question of risk assessment and management in general psychiatric hospitals (34). Optimizing specific training for mental health caregivers regarding the assessment of risk for violence, which is currently scarce in France, and developing prevention measures are thus key factors in improving this issue and should be a priority in general psychiatric hospitals.

Several limitations of the present work should be acknowledged. First, we had access to data only on people found NCRMD referred to psychiatric hospitalization. We thus did not include people found NCRMD who were discharged without any psychiatric hospitalization. However, in our experience, these situations are particularly rare in France. Second, we had access only to health data from the PMSI, and no information about the severity of the offenses or the patients' criminal history was therefore available in this database. It was not possible to study offenses (and incarcerations) prior to patients' admission to and after their discharge from their NCRMD psychiatric hospitalization. Third, for the same reason, we presented the number of hospital admissions for people found NCRMD each year, but it was not possible to calculate the ratio of people found NCRMD to the total number of convictions each year in France. Fourth, the description of the patients was limited to their age, sex and diagnosis, making it impossible to investigate the clinical characteristics associated with the longest lengths of stay or hospitalizations in maximum-security units. The causes of death of the deceased individuals were not available either. Fifth, we only had access to the data from 2011 to 2020; therefore, it was not possible to extend the study period beyond this time frame.

In conclusion, we conducted the first study investigating psychiatric hospital treatment of people found NCRMD in France. This work, performed over a 10-year study period (2011–2020), provides some information on the very poorly known population of individuals found NCRMD. However, there is an urgent need for further studies to better identify the clinical characteristics of these patients and to provide individualized and optimized management to this population (22).

## Data Availability Statement

The original contributions presented in the study are included in the article/Supplementary Materials, further inquiries can be directed to the corresponding author/s.

## Author Contributions

TF, MB, and AL participated in the conception and design of the study. MB and AL participated in the acquisition of data and performed the analyses. TF wrote the first draft of the manuscript. All authors participated in the writing and revision of the successive drafts of the manuscript and approved the final version.

## Conflict of Interest

The authors declare that the research was conducted in the absence of any commercial or financial relationships that could be construed as a potential conflict of interest.

## Publisher's Note

All claims expressed in this article are solely those of the authors and do not necessarily represent those of their affiliated organizations, or those of the publisher, the editors and the reviewers. Any product that may be evaluated in this article, or claim that may be made by its manufacturer, is not guaranteed or endorsed by the publisher.
