# Supplementary material for: Psychiatric Hospitalizations of People Found Not Criminally Responsible on Account of Mental Disorder in France: A Ten-Year Retrospective Study (2011–2020)
Source: Front Psychiatry. 2022 Apr 5;13:812790. doi: 10.3389/fpsyt.2022.812790 (PMC9016162; doi:10.3389/fpsyt.2022.812790)
Supplement: Supplementary file 1 [file Table_1.DOCX]

Supplementary Material

**Supplementary Table 1.** Number of individuals found not criminally responsible on account of mental disorder and referred to psychiatric hospitalization in France (2011-2020).

| Year | Number of distinct patients | Number of admissions | Number of discharges |
| --- | --- | --- | --- |
| 2011 | 710 | 263 | 149 |
| 2012 | 869 | 308 | 254 |
| 2013 | 906 | 291 | 239 |
| 2014 | 876 | 209 | 210 |
| 2015 | 885 | 219 | 201 |
| 2016 | 922 | 238 | 199 |
| 2017 | 983 | 260 | 251 |
| 2018 | 1003 | 271 | 256 |
| 2019 | 1034 | 287 | 329 |
| 2020 | 932 | 227 | 378 |

**Supplementary Table 2.** Principal diagnoses that motivated the hospitalization for lack of criminal responsibility among patients found not criminally responsible on account of mental disorder admitted after 2016, with or without a previous psychiatric hospitalization during the previous 5 years.

|  | With hospitalization before 2016 (N=930) | Without hospitalization before 2016 (N=353) |
| --- | --- | --- |
| *F2 - Schizophrenia, schizotypal, delusional, and other non-mood psychotic disorders* | 582 (62.5%) | 173 (49.0%) |
| *F6 - Disorders of adult personality and behaviour* | 82 (8.8%) | 34 (9.6%) |
| *F3 - Mood (affective) disorders* | 67 (7.2%) | 29 (8.2%) |
| *F1 - Mental and behavioural disorders due to psychoactive substance use* | 52 (5.6%) | 15 (4.2%) |
| *F4 - Anxiety, dissociative, stress-related, somatoform and other nonpsychotic mental disorders* | 29 (3.1%) | 17 (4.8%) |
| *Other diagnoses* | 89 (9.6%) | 52 (14.7%) |
| *Missing diagnosis* | 29 (3.1%) | 33 (9.3%) |
